# Comparison of the caries-protective effect of fluoride varnish with treatment as usual in nursery school attendees receiving preventive oral health support through the Childsmile oral health improvement programme — the Protecting Teeth@3 Study: a randomised controlled trial

**DOI:** 10.1186/s12903-015-0146-z

**Published:** 2015-12-18

**Authors:** William Wright, Stephen Turner, Yulia Anopa, Emma McIntosh, Olivia Wu, David I. Conway, Lorna M. D. Macpherson, Alex D. McMahon

**Affiliations:** Community Oral Health, University of Glasgow Dental School, 378 Sauchiehall Street, Glasgow, G2 3JZ Scotland, UK; Health Economic and Health Technology Assessment, Institute of Health & Wellbeing, University of Glasgow, 1 Lilybank Gardens, Glasgow, G12 8RZ Scotland, UK

**Keywords:** Duraphat®, Fluoride Varnish, Childsmile, Nursery School, Effectiveness, Caries, Prevention

## Abstract

**Background:**

The Scottish Government set out its policy on addressing the poor oral health of Scottish children in 2005. This led to the establishment of Childsmile, a national programme designed to improve the oral health of children in Scotland. One element of the programme promotes daily tooth brushing in all nurseries in Scotland (Childsmile Core). A second targeted component (Childsmile Nursery) offers twice-yearly application of fluoride varnish to children attending nurseries in deprived areas. Studies suggest that fluoride varnish application can reduce caries in both adult and child populations. This trial aims to explore the effectiveness and cost-effectiveness of additional preventive value fluoride varnish application compared to Childsmile Core.

**Methods/Design:**

The Protecting Teeth@3 Study is an ongoing 2 year parallel group randomised treatment as usual controlled trial. Three-year-old children attending the ante pre-school year are randomised (1:1) to the intervention arm (fluoride varnish & treatment as usual) or the control arm (treatment as usual). Children in the intervention arm will have Duraphat® fluoride varnish painted on the primary tooth surfaces and will continue to receive treatment as usual: the core Childsmile Nursery intervention. Children in the treatment as usual arm will receive the same series of contacts, without the application of varnish and will also continue with the Childsmile Core intervention. Interventions are undertaken by Childsmile trained extended duty dental nurses at six-monthly intervals. Participants receive a baseline dental inspection in nursery and an endpoint inspection in Primary 1 at the age of 5 years old.

We will use primary and secondary outcome measures to compare the effectiveness of Duraphat® fluoride varnish plus treatment as usual with treatment as usual only in preventing any further dental decay. We will also undertake a full economic evaluation of the trial.

**Trial registration:**

This study is registered at ClinicalTrials.gov. Number: NCT01674933 (24 August 2012).

**Electronic supplementary material:**

The online version of this article (doi:10.1186/s12903-015-0146-z) contains supplementary material, which is available to authorized users.

## Background

This study will be performed according to the Research Governance Framework for Health and Community Care [6] and The Medicines for Human Use (Clinical Trials) Regulations [24]) SI 2004:1031 (as amended), and World Medical Association Declaration of Helsinki: Ethical principles for medical research involving human subjects 1964 (as amended). Amendment number 4, Protocol version V2.3, 31st July 2015. Duraphat has been considered to be an Investigational Medicinal Product for the purposes of this trial. The notification from the MHRA is shown in Additional file 1; the study has been given the EUDRACT number 2012-002287-26. All investigators and key trial personnel will complete biennial Good Clinical Practice training.

In 2005 the Scottish Government set out its policy on addressing the poor oral health of Scottish children. A series of reports had highlighted persistently high rates of dental caries with significant inequalities in oral health [16], low rates of NHS dental registration for young children (35 % of 0–2 year-olds in 2004) [17], and extremely limited preventive activity [18].

The 2005 Scottish Government Report “An Action Plan for Improving Oral Health and Modernising Dental Services in Scotland” announced what was to become the Childsmile oral health improvement programme [19]. The aim was to shift the balance of care towards more preventive and anticipatory care and target the early years in an attempt to promote health improvement from a young age. Programme development was founded on the principles outlined in the Ottawa Charter for Health Promotion [30].

Two Childsmile Demonstration Programmes were subsequently established in 2006, one in the East and one in the West of Scotland, with a targeted approach to improving the oral health of young children. Both were to run initially for 3 years, in order to allow the Programmes to evolve as a result of ongoing monitoring, evaluation, and stakeholder feedback. The Programmes complemented a national toothpaste/toothbrushing scheme set up a few years previously, whereby free toothpaste/toothbrush packs are distributed to every child in Scotland on at least six occasions during their first 5 years and free daily toothbrushing offered to every 3 and 4 years old child attending nursery schools in Scotland. The toothbrushing programme is also available to primary one and two children in schools situated in disadvantaged areas of National Health Service (NHS) Boards across the country. This programme, which became Childsmile Core, built on pre-existing local NHS Board-based toothbrushing initiatives, and enabled standardisation across the country with national procurement of the toothbrushing supplies and the publication and implementation of national standards for toothbrushing programmes [12, 13].

In nursery schools - the setting for the present study - Childsmile Core promotes daily tooth brushing, backed up with free dental packs containing fluoride toothpaste (circa 1000 ppm F), toothbrushes and advice leaflets, fruit and freely available water. Children in nurseries in deprived areas are also offered twice-yearly application of fluoride varnish from the time they start nursery under the Childsmile Nursery programme. In the period December 2006 to July 2011 168,000 Duraphat applications were completed under this programme, with no reported significant adverse events.

However, it is important to ascertain the additional preventive value fluoride varnish application in nursery schools may bestow over and above that gained from the Childsmile Core activities described above. There is some evidence from quasi-experimental data at the population level that the Childsmile Core intervention on its own has indeed improved the oral health of children in the Greater Glasgow area [2, 3]. The study will establish whether fluoride varnish will deliver benefits which are additional to those of the Childsmile Core Programme.

A systematic Cochrane Review of fluoride varnish application (Marinho et al. [9] concluded that this treatment reduced worsening of caries (known as a 'prevention fraction') in the primary dentition by 33 %, as measured by the 'd_3_mfs' score for deciduous teeth (number of decayed (at the dentine level), missing and filled surfaces). At the 'd_3_mft' level (decayed missing and filled teeth), the amount of prevented disease was possibly larger with an estimate of 53 %. Most of the studies included in the review were from populations with regular use of fluoride treatments, especially toothpaste.

On the one hand it is known that many deprived children in Scotland have not been using fluoride toothpaste in the past, but on the other hand the Childsmile programme should be addressing this problem. Perhaps a 33–53 % reduction in worsening of dmfs/dmft is a reasonable estimate of how much fluoride varnishes could achieve in a deprived population in the West of Scotland. However, the Cochrane review was only based upon nine trials, and only two of these looked at the deciduous teeth of 3 and 4 year olds. The 'numbers needed to treat' varied from 3.7 in low caries populations to 1.6 in higher caries populations.

A number of relevant studies have been published since the 2002 review. A randomised controlled trial by Skold et al. [21] involved Swedish schoolchildren being assigned different frequencies of varnish treatment, across three different levels of dental risk including a 'high risk' group. This study demonstrated a prevention fraction for varnish at six-monthly intervals of 69 % in high risk areas, 66 % in medium risk areas, and only 20 % in low risk areas. Although of great interest the study is of secondary school children aged 13 to 16 years, so does not answer the specific questions addressed in the present study. A German study reported by Borutta et al. [4]) appears to be closer to the proposed study. The aim was to assess the caries inhibition effect of fluoride varnishes among preschool children with high caries risk though an examiner-blind, clinically controlled 2-year study with 200 randomly selected 2- to 4-year-olds. As with the present study, four varnish applications were offered. The study reports a caries reduction level of 57 % for the fluoride varnish compared with controls. However there is no reference to the study taking place within an established prevention programme as represented by Childsmile Nursery in the present study.

A third RCT [7] investigated the preventive effect of fluoride varnish in primary school children aged around 7 years old. Randomisation was at the school year level with either Primary 2 or Primary 3 receiving fluoride varnish treatment. The percentage of children with increased caries was high in both intervention (53 %) and 'treatment as usual' group (50 %). Weaknesses in this study include the 'clustered within the same school' design (which could have encouraged compensatory action to improve dental health in control classes), blinding limited to the evaluator, and difficulties recruiting children with a high risk of caries.

A recent trial involving schoolchildren in the North West of England [10] also failed to find any evidence of effectiveness of fluoride varnish in a public health programme. However, the North West England trial was undertaken in older children (aged 7–8 years old), while the present study focuses on 3 and 4 year olds. In addition the small amount of varnish used in the North West England trial (3 applications of 22,600 ppm fluoride varnish per year for 3 years) applied to permanent molars may have been insufficient to provide effective dental caries prevention. Finally, the present study takes place within the Childsmile programme of supervised nursery toothbrushing for children from more deprived areas who may be more at risk of developing caries.

### Study rationale – hypothesis

In nursery schools - the main setting for the present study – the Scottish Government’s Childsmile Core programme promotes daily tooth brushing, backed up with free dental packs containing fluoride toothpaste (circa 1000 ppm F), toothbrushes and advice leaflets, fruit and freely available water. Children in nurseries in deprived areas are also offered twice-yearly application of fluoride varnish from the time they start nursery. It is important to ascertain the additional preventive value this may bestow over and above that gained from the Childsmile Core activities described above. The study will establish whether fluoride varnish will deliver benefits which are additional to those of the Childsmile Core Programme.

The null hypothesis is therefore that the dental health (measured by d_3_mft score) of children in the fluoride varnish arm of the study is no better after 18 months of varnish application than that of the children in the TAU arm. The alternative to the null hypothesis is that there will be a significantly lower increase in the d_3_mft scores of children in the fluoride varnish arm than that found in the d_3_mft of children in the TAU arm.

### Study objectives

The objective of this study is to compare the effectiveness of Duraphat fluoride varnish plus treatment as usual (TAU) with TAU only in preventing any further dental decay. The primary endpoint for each individual child is whether or not there has been any occurrence of new caries lesions over the 2 year period, as measured by any increase in d_3_mft at 2 years of follow up compared to the d_3_mft at baseline (d_3_mft is dental decay as measured by the dmft scale in the dentine).

## Methods/Design

**Fig. 1:**
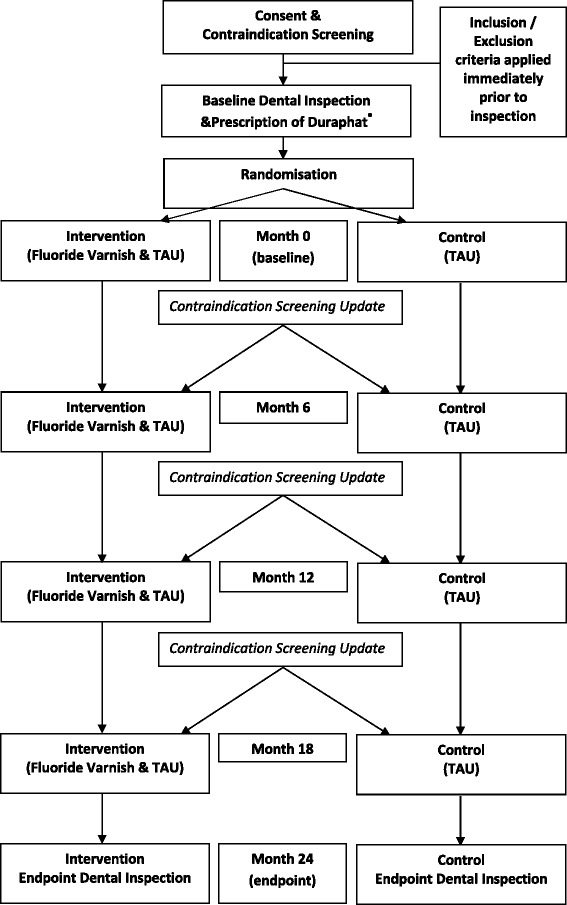
Flow diagram of the PT@3 Study

This protocol was peer reviewed by the national evaluation board of the Childsmile Programme, which was set up by the funder (Scottish Government) to have oversight of all research and evaluation that is associated with the programme. The design is described diagramatically in Fig. 1.

### Study population

Eight hundred five children will be recruited into each of the two arms of the study. They will be identified in approximately 50 nursery schools in the NHS Lothian, Greater Glasgow and Clyde, Fife and Tayside Health Board areas. Nursery schools selected for this study will serve children likely to be at a higher than average risk of caries (e.g., because the nursery school serves a population from postcode areas with a SIMD score indicating higher social deprivation). The sample is 3-year-olds attending participating nursery schools. Eligibility regarding absence of contraindications will be assessed by enquiries to parents and schools prior to study entry. Routine Childsmile information on fluoride varnish will be used, as well as the study information from a specially designed ‘participant information sheet’ (PIS).

Eligible participants will be randomised (1:1) to receive Duraphat ® fluoride varnish plus TAU or TAU only. Randomisation will follow the baseline dental examination by a study dental practitioner, and will take place via a telephone call to the Interactive Voice Response System (IRVS) at the Robertson Centre for Biostatistics, University of Glasgow. Blocks of four and two will be used within each nursery school, so that for every four/two children each of the two treatments will be allocated equally. Because the blocking is done at each nursery separately we will not necessarily complete the last block at each of the schools (i.e., when the target number of subjects in each treatment group has been reached). Members of the Extended Duty Dental Nurse (EDDN) teams undertake the randomisation of participants and are allocated a Personal Identification Number (PIN) by the RCB and trained in the use of the IVRS.

### Inclusion criteria

- Provision of a signed informed consent form from a parent or legal guardian.
- Children in the first year of nursery school (known as the ‘ante pre-school year’).
- Every eligible child in participating nurseries will be invited to join the study, irrespective of the SIMD ranking of their own postcode.
- Children with or without pre-existing cavities, as the cavity can be treated through the usual primary care dental service (i.e., as part of ‘treatment as usual’).

### Exclusion criteria

- Children with contraindications for the Duraphat varnish i.e., hypersensitivity to colophony and/or any other constituents.
- A history of bronchial asthma requiring hospitalisation.
- History of allergic episodes requiring hospital admission.

### Identification of participants and consent

Once the head teacher has given permission, an information sheet and consent form will be given or sent to the parents or guardians of every ante pre-school child. The participant information sheet makes clear the duration of the trial and that participation is voluntary and that the parent or guardian can withdraw their child at any point. Child home postcodes from the school registration rolls will be linked to the Scottish Index of Multiple Deprivation (SIMD) to ensure any potential participation bias can be analysed, and to ensure outcomes can be assessed by deprivation.

Children will not be approached for consent/assent due to their young age. Informed consent will be obtained directly from the parents/guardians by members of the study team at their child’s nursery, at the consenting visit which will take place at least 24 h after the distribution of the PIS. Trial staff will also ask consenting parents and guardians to complete a contraindications checklist (adapted from the Childsmile Consent Form for Toothbrushing & Fluoride Varnish [version 7.1]). The checklist will screen children for the contraindications for Duraphat® varnish (listed above). All checklists with information on possible contraindications will be assessed to decide if the child should be excluded from the trial. On the day of the dental inspection any child showing distress or verbal or non-verbal signs of extreme reluctance will be excluded from the study if the dentists feel that continuing with the inspection would cause the child further distress.

### Withdrawal of subjects

Immediately prior to the 6, 12, and 18 Month interventions, parents/guardians will be requested to update information on contraindications to Duraphat® varnish. Parents/guardians will be sent a self-complete version of the Contraindications checklist (adapted from the Childsmile Consent Form for Toothbrushing & Fluoride Varnish [version 7.1]) and a covering letter. The checklist will again screen children for the contraindications for Duraphat® varnish listed above. The covering letter makes it clear that the checklist must be returned if the child has developed any contraindications in the period following the previous intervention, so we can exclude that child from the intervention. All returned checklists will be assessed by the trial dentist who will decide if the reported contraindication for Duraphat® varnish requires that the child be withdrawn from the study.

Parents who withdraw their child will be asked, if appropriate, to agree to their having the end-of-study dental examination, in order to make best use of information already collected on that child. Children who leave a trial nursery school during the study, but remain in the local area, and go on to attend a local primary school will receive an endpoint dental inspection. The parents/guardians of these children will be informed of the inspection.

Any adverse reactions to the fluoride varnish (e.g., mucositis, allergic reaction etc.), whether noted by Childsmile staff or reported by parents or school, will be entered into the Case Report Form by the study dental nurse or dental health support worker. All recorded adverse reactions will be reviewed by the site Principal Investigator. Details of adverse reactions will be assessed against the criteria for expedited pharmacovigilance reporting to the MHRA and Research Ethics Committee. If any immediate adverse reaction is suspected, the varnish can be easily removed by toothbrushing, rinsing and spitting. Any child with a suspected immediate adverse reaction will be permanently withdrawn from the study.

### Study schedule

Up to six visits are involved for children who are retained throughout the study. The first will be to complete the baseline dental inspection. This is followed by randomisation and the first of four intervention/TAU visits by the study Childsmile team to the nursery school, spaced roughly six months apart. The final visit is to undertake an endpoint dental inspection to re-inspect the child in year 1 of their primary school. Neither children nor parents are required to make special visits to a clinic or other study base. Where possible, visits 1 and 2 will be combined: i.e., if logistics allow, the first varnish/TAU intervention will be completed after the baseline dental examination and the subsequent randomisation.

#### Visit 1/visit 2: Examination/randomisation/0 month intervention visit(s)

At this visit study staff will confirm eligibility before the baseline dental examination is carried out by a study dental practitioner (e.g., signed consent form is in place, enquiry form regarding contraindications is complete and the child has no obvious temporary infections or injuries which would lead to exclusion on the day). Completion of CRF containing dental examination data.

All children receive treatment as usual (TAU), namely supervised toothbrushing in the nursery. Randomisation will take place via a telephone call to the IRVS at the Robertson Centre for Biostatistics. As soon as possible following randomisation, the study Childsmile team will perform a brief oral check, depending on randomisation they either get a “sham” fluoride varnish application (applicator brushing teeth with no fluoride varnish on it) or they get the active treatment involving application of fluoride varnish. The dental nurse will record treatment arm in order to ensure consistency of treatment throughout the sequence of visits.

#### Visit 3: 6 Month (+/- 3 months) intervention visit to nursery school

In the week prior to the visit parents will be sent the contraindications checklist and covering letter (detailed above). If the parents are aware of any contraindications this checklist will be returned to the nursery school. On arrival at the nursery school the study dental team will ascertain if any checklists have been returned. If no exclusion criteria are identified, the study dental team will again either apply fluoride varnish, or deliver TAU, including a brief oral check, as dictated by the original study arm allocation. As before, children with temporary conditions (e.g., cold sores, abrasions, or systemic illnesses) will not have FV applied, but will remain in the study. Children absent at this or subsequent visits will also continue in the study unless actively withdrawn by a parent or following assessment of any adverse reaction.

#### Visit 4: 12 Month (+/- 3 months) intervention visit to nursery school

Procedures as for Visit 3 above.

#### Visit 5: 18 Month (+/- 3 months) intervention visit to nursery school

Procedures as for Visit 3 above. If necessary, this visit may take place in the first term of the child’s first year in Primary school. Relevant school staff will be informed if this required.

#### Visit 6: 24 Month (+/- 3 months) end of study dental examination visit to primary school

This visit will be conducted when participating children are in Primary One, and 5 years old on average. The final dental examination will be carried out, regardless of whether or not the sequence of varnish applications or TAU contacts had been interrupted or discontinued for any reason.

#### Unexpected end of study visit

In the event of a child having to leave the study due to withdrawn consent, or moving home and so on, the head teacher of the school will inform the study team. If the parent agrees, arrangements will be made to complete the end-of-study dental inspection in order to maintain that child in the study database.

### Study outcome measures

#### Primary outcome measure

The primary outcome measure is d_3_mft at 2 years of follow up compared to the d_3_mft at baseline (d_3_mft is dental decay as measured by the number of: teeth affected by decay into the dentine; missing teeth; and filled teeth).

#### Secondary outcome measures

##### d_3_mfs

The d_3_mfs scale is a count of affected surfaces rather than teeth. The d_3_mft and d_3_mfs measures will be obtained through detailed dental examinations which follow the protocol adopted for the NHS Scotland National Dental Inspection Programme (NDIP), which involve P1 and P7 children. The examinations will be conducted by NDIP dentists following a training and calibration exercise. In addition, dental decay outcomes will be assessed by deprivation SIMD strata.

##### Child records of dental treatment and attendance

With parental informed consent, the children’s records of dental treatment and attendance held by Information and Statistics Division, NHS Scotland, will be examined to establish a profile of contact with and treatment by dental services in the two arms of the study.

##### Quality of life

In addition to clinical data, the trial will also collect data on children’s’ general and oral health and quality of life using a battery of questionnaires, distributed at three points in the trial: at baseline, 12 months and 24 months. Parents/guardians of participants will be asked to complete:

- The Child Health Utility 9D for under 5-year-old children (CHU9D) – parental proxy questionnaire ([23, 27], and Katherine Stevens, personal communication, May-June 2014) (Additional file 2);
- The Paediatric Quality of Life Inventory (PedsQL) – the PedsQL 4.0 Generic Core Scale Parental Report for Toddlers (ages 2–4 years) [29, 22, 30] (Additional file 3);
- The Scale of Oral Health Outcomes for 5-year-old children (SOHO-5), parental proxy questionnaire ([25, 26], and Georgios Tsakos personal communication, May 2014) (Additional file 4).

##### Health and dental care services resource use

A questionnaire (Additional file 5) designed to elicit information on uptake of health and dental care services and medication use by the child within the past 12 months will also be distributed in the quality of life questionnaire pack at each of the three rounds.

##### Subjective global transition judgement question

Parents/guardians will be asked to answer two subjective global transition judgement questions in the questionnaire packs (Additional file 6) distributed at 12 and 24 months. This will be used as a method to assess the QoL instruments’ responsiveness to change [1]. The questionnaire packs will be distributed to the families via the nurseries or posted to the family’s home address.

### Follow-up procedures parental questionnaire survey

A number of well-established techniques to enhance response rates will be used. These are:

- the cover page (Additional file 7) for the questionnaire pack sent at baseline offer parents/guardians the opportunity to enter a prize draw for an iPad. This offer will be repeated for each of the two subsequent rounds of the survey;
- there will be an unconditional incentive in the form of a Childsmile branded pen and a page of Childsmile stickers (for the child) contained within the questionnaire pack;
- a sense of participation in the study will be encouraged by the distribution of Christmas cards and/or a study newsletter updating on the progress of the study, and encouraging completion of the parental questionnaires;
- parents who had previously provided a mobile telephone number will receive a text reminder if they have not returned the questionnaires within 1 week;
- at the end of the second week a second copy of the questionnaire pack will be mailed to any non-responders directly to their home address or distributed via the child’s nursery;
- at the end of the third week the non-responder parent will be contacted by telephone to remind them to complete the questionnaires.

The techniques described above have been adapted from those used by the Seal or Varnish trial [5] run by South East Wales Trials Unit/Cardiff University.

### Data linkage

In order to be able to link the trial outcome measures by child with the children’s records of dental treatment and attendance held by Information and Statistics Division, NHS Scotland, we will request, at the beginning of the trial, a full home address from the parents/guardians of trial participants.

### Laboratory tests

None.

### Assessment of safety

As part of the recruitment process, parents will be asked about contraindications for fluoride varnish. The most common of these are a history of hospitalisation for bronchial asthma or allergic reactions. Other contraindications are: hypersensitivity to colophony and/or any other fluoride varnish constituents.

Temporary conditions such as ulcerative gingivitis and stomatitis will be checked on the day of the application, following the normal Childsmile procedure detailed below. This procedure is within the clinical remit of the Extended Duty Dental Nurses employed to deliver the Childsmile Fluoride Varnish programme, and includes the following steps:

- check the skin of the face and around the mouth for abnormalities (spots, inflammation, swelling etc.)
- check the lips for lesions/infections.
- check the inner cheeks and the insides of the lips
- check the upper and lower surfaces of the tongue.
- Children showing obvious signs of systemic illness (e.g., colds, flu) or any abnormality of the face, lips or soft tissues of the mouth should be excluded on the day from Fluoride Varnish application.
- The teeth and gums should be checked for signs of infection in a systematic order.

#### Adverse reaction reporting

If there are any immediate adverse reactions to the Fluoride Varnish (e.g., mucositis, allergy etc.) the product will be removed by toothbrushing and rinsing, following the Childsmile local protocol. The possible adverse reaction may be noticed immediately by the dental team or later by the teachers or parents. Any such reports will be transmitted by the Childsmile study team to the family dentist, the Childsmile coordinator and the local Principal Investigator. This information will be updated on the child’s notes accordingly.

### Investigational drug information

Children who are eligible for the study will be randomised to receive fluoride varnish treatment as Duraphat® Dental Suspension. Further details of treatment schedules are given below.

#### Treatment schedule

At each treatment, a total volume of 0.25 ml of Duraphat® Dental Suspension will be applied to the teeth in children randomised to the intervention treatment. Each 1 ml of Duraphat® Dental Suspension contains 50 mg sodium fluoride which is equivalent to 22.6 mg of fluoride. The protocol treatments will be applied at six monthly intervals for 18 months i.e., baseline, and at 6 months, 12 months and finally 18 months post-baseline (all timings +/- 3 months).

#### Rationale for chosen treatment schedule

The treatment schedule chosen is in line with current practice guidelines (Guideline 83. [20]) and the approved summary of product characteristics.

A 0.25 ml dose of Duraphat® dental suspension contains 5.6 mg of fluoride. The toxic dose of fluoride ingestion is estimated at 75 mg for an average sized 3 year old weighing 15 kg, so such a child would have to swallow 2 whole cartridges to ingest a toxic amount. Any child suspected of swallowing excessive levels should be given lots of milk to drink and transferred to the local A&E Department for a gastric lavage [9].

A child in the study may receive fluoride varnish up to a maximum of four times a year (twice in study applications and twice from their own dentist if their practice is participating in their Childsmile Practice programme.) This represents a total dose of 22.6 mg of fluoride spread over four applications per year, and is well within the Scottish Dental Clinical Effectiveness Programme’s (SDCEP) clinical guidelines [15] and is within the safe limits for both acute toxicity levels and chronic ingestion resulting in fluorosis. Even if the child were to receive two doses on the same day, there would be no risk of toxicity as two doses would give the child 11.3 mg of available fluoride, still well within the dose safety margin. There would also be very little chance of fluorosis as, after the age of 4 years, most of the adult teeth have already calcified. A dose of 25 mg four times per year is within the SDCEP’s clinical guidelines [15].

#### Investigational product administration

The procedure will follow the standard practice of the Childsmile Nursery programme [13] and will be detailed in full in the study management procedures. An outline of the procedure is given below.

##### Safety assessment prior to treatment

A safety assessment procedure will be carried out by the team prior to each treatment application. The safety assessment reduces the possibility of children with oral/facial infections being included. Any child with any abnormality of the lips, face or soft tissues of the mouth will be excluded from fluoride varnish treatment scheduled for that day. Children who are showing obvious signs of systemic illness e.g., colds, 'flu, chicken pox etc. will also be excluded on that day. An extra-oral and intra-oral assessment will be conducted. Thereafter, the teeth and gums will be checked for signs of infection starting with the upper right and then moving to the upper left, lower right and finally the lower left. If the risk assessment is negative the fluoride varnish will be applied. Children who have signs of decay will still have the fluoride varnish applied as it may help protect from further decay and it will familiarise the child to dental treatment.

##### Treatment

Starting with the upper right side, the cheek will be retracted with a finger or mirror and the canine and molars dried with a cotton roll. With the cotton roll in place, a small amount of Fluoride Varnish will be applied to the contact points of the canine and molars and to the occlusal surfaces of the molars. The same procedure will be repeated for the upper left quadrant and lower quadrants. The remaining varnish will be applied to the labial surfaces of the canines and incisors. The dental nurse will then complete a check to ensure that all equipment is removed from the mouth and disposed of in line with current procedures.

If the child becomes upset or protests during any part of the procedure, then the procedure will be halted, and only resumed if the child can be reassured and put at ease. If any immediate adverse reaction is suspected, the varnish can be easily removed by toothbrushing, rinsing and spitting. Children who miss one or more of the treatments are still eligible to receive any remaining treatments. Children with an incomplete record of treatments or contacts will be retained in the study, even if all treatments and contacts are missed.

### Study supplies

#### Supply of study treatment

Commercially available UK supplies of Duraphat® Dental Suspension will be used in the study. All supplies for use in the study will be sourced from via normal NHS supply mechanisms. There will be no study specific supplies.

Duraphat® Dental Suspension is presented in 10 ml aluminium tubes. As per current standard practice, each tube will be used for multiple participants. In order to prevent any potential cross-contamination, a single 0.25 ml dose will be measured out by the dental nurse prior to the start of the procedure and the resealed tube will be returned to either a locked cupboard or transport box. Any Duraphat® Dental Suspension which remains at the end of 7 days will not be used for study purposes.

#### Labelling of study treatment

There will be no study specific labelling. All supplies to be used in the study will be UK licensed stock prescribed by a dentist and labelled in accordance with current regulatory requirements for Prescription Only Medicines including instructions for use, expiry date and batch details. The investigational product will be used in accordance with the Summary of Product Characteristics for multiple study participants within a clinic type setting under a dentist prescription and will not be dispensed for use by an individual participant. To ensure traceability, the batch number will be recorded at each participant treatment.

#### Storage of study treatments

As this is a phase IV study of an established treatment intervention conducted as part of a national programme, continuous temperature monitoring of the investigational medicinal product would be logistically difficult to achieve and, from a risk perspective based on the clinical use of the medicine, in excess of current practice standards. However in an effort to balance any potential risks associated with limited exposure to temperatures outwith the recommended storage temperature of 25oC, opened tubes will be discarded after 7 days.

#### Drug accountability

In order to ensure full traceability, the batch number used for each treatment of every individual participant will be recorded on the Case Report Form. Details will also be recorded on the CRF where there is only partial application.

### Pharmacovigilance

#### Definitions of adverse events

##### Adverse Event (AE)

Any untoward medical occurrence in a subject to whom a medicinal product has been administered, including occurrences which are not necessarily caused by or related to that product.

##### Adverse Reaction (AR)

Any untoward and unintended response in a subject to an investigational medicinal product which is related to any dose administered to that subject.

#### Serious Adverse Event (SAE) or Serious Adverse Reaction (SAR)

Any adverse event or adverse reaction that:

- results in death,
- is life threatening,
- requires hospitalisation or prolongation of existing hospitalisation,
- results in persistent or significant disability or incapacity,
- consists of a congenital anomaly or birth defect,
- is otherwise considered medically significant by the investigator,
- Important adverse events/reactions that are not immediately life-threatening or do not result in death or hospitalisation but may jeopardise the subject or may require intervention to prevent one of the other outcomes listed in the definition above.

### Suspected Serious Adverse Reaction (SSAR)

Any adverse reaction that is classed in nature as serious and which is consistent with the information about the medicinal product in question set out in the summary of product characteristics (SmPC) or the Investigator’s Brochure (IB).

#### Suspected Unexpected Serious Adverse Reaction (SUSAR)

Any adverse reaction that is classed in nature as serious and which is not consistent with the information about the medicinal product in question set out in the SmPC or the Investigator’s Brochure (IB).

#### Detection, recording and reporting of Adverse Events

It is not proposed to collect details of all adverse events occurring in this study. Only information on adverse reactions to the IMP will be collected. The applications are spaced approximately 6 months apart, and it is inappropriate and unnecessary to attempt to collect information throughout each 6 month period on events unrelated to the varnish application, when it is known what the very specific, immediate and limited range of possible adverse reactions may be. Any adverse reaction is extremely likely to be seen within a maximum of 24 h post application. Any adverse events reported by parents during that time will be recorded and evaluated by the Childsmile coordinator and the local Principal Investigator irrespective of whether it constitutes a possible reaction or an unconnected event.

All Adverse reactions must be recorded, notified, assessed, reported, analysed and managed in accordance with the Medicines for Human Use (Clinical Trials) Regulations 2004 (as amended) and this protocol. (See Safety Flow chart- Additional file 8).

Any adverse reactions to the fluoride varnish (e.g., mucositis, allergy etc.), whether noted by Childsmile staff or reported to Childsmile staff by parents or the nursery school, will cause either the study dental nurse or dental health support worker to record details in the Case Report Form and to inform the site Principal Investigator to review such events. The parents or guardian may refer to the fluoride varnish aftercare leaflet for details of the local Childsmile Coordinators acting as the contact point regarding any possible adverse reaction.

The site Principal Investigator will assess the severity and seriousness of such events, and re-assess their relatedness to the administration of Duraphat varnish. Events deemed to be serious and related to the administration of the varnish will be classified as serious adverse reactions (SARs). SARs are to be reported by the site to the Sponsor’s Pharmacovigilance Office within 24 h of awareness of such event for assessment against the criteria of expectedness. The management of SARs is detailed below.

The expectedness of an adverse reaction is assessed against the approved Reference Safety Information i.e., the list of expected reactions detailed in the SmPC.

The following is noted in the SmPC:

- “In subjects with a tendency to allergic reactions, oedematous swelling of the oral mucosa has been observed in exceptional cases, especially after extensive application. If necessary, the dental suspension layer can easily be removed from the mouth by brushing and rinsing. Ulcerative gingivitis and stomatitis have been reported by sensitive individuals.
- In rare cases, asthma attacks may occur in participants who have bronchial asthma.
- In participants with gastric sensitivity, retching may exceptionally occur after a high dosage and extensive application.”

The expectedness of a SAR will be determined by the Chief Investigator following discussion with the site Principal Investigator.

#### Severity

This should be assessed and described using the following categories:

- mild-awareness of event but easily tolerated,
- moderate-discomfort enough to cause some interference with usual activity,
- severe-inability to carry out usual activity.

Any AE that is assessed as serious, is suspected of having a causal relationship to the trial medication and is unexpected is a SUSAR and will require expedited reporting to the MHRA/Ethics Committee as detailed below. Should an SAR be deemed to be unexpected, the Pharmacovigilance Office will liaise with the Chief Investigator to complete the expedited safety report.

Adverse reactions and Serious Adverse Reactions that occur up to 24 h after Fluoride varnish application will be reported.

Any adverse reactions present at the last application must be followed up until the event is resolved. The participant is considered to have completed the study EITHER after the completion of the last visit or contact (e.g., phone contact with the site Principal Investigator), OR after the last application of fluoride varnish, whichever is later; OR the participant can no longer comply with the requirements for any further study visits or evaluations.

#### Reporting of serious adverse reactions

All SARs (as detailed above) arising during the study will be reported by the site Principal Investigator or site Childsmile study team following review by the Principal Investigator to the sponsor (Glasgow Clinical Trials Unit (GCTU) Pharmacovigilance (PV) Office) as soon as reasonably practicable and in any event within 24 h of first becoming aware of the event. Any follow up information should also be reported.

A Serious Adverse Event form is completed and forwarded to the Pharmacovigilance Office.

- The GCTU Generic CTIMP SAE form is downloaded from www.glasgowctu.org, printed off, completed and signed. The form is then faxed to the Pharmacovigilance Office on 0141 357 5588. A copy is placed in the Study Site File.
- If necessary a verbal report can be given by contacting the Pharmacovigilance Office on 0141 330 4744. This must be followed up as soon as possible with a signed written report.

Serious adverse reaction details will be transferred to the Glasgow Pharmacovigilance database.

All SUSARS must be reported in an expedited fashion to the MHRA and Ethics Committee

- Fatal or life threatening SUSARs: not later than 7 days after the sponsor had information that the case fulfilled the criteria for a fatal or life threatening SUSAR, and any follow up information within a further 8 days.
- All other SUSARs: not later than 15 days after the sponsor had information that the case fulfilled the criteria for a SUSAR

The Pharmacovigilance office will report SUSARs to the MHRA on behalf of the Chief Investigator via the MHRA eSUSAR reporting system and to Ethics committee by email.

#### Annual safety reporting

An annual safety report is required to be submitted to MHRA and REC within 60 days of the anniversary of the issue of the Clinical Trials Authorisation. The Chief Investigator will submit this report in liaison with the Pharmacovigilance Office.

### Statistics and data analysis

#### Statistical analysis plan

The study will have a comprehensive Statistical Analysis Plan, which will govern all statistical aspects of the study, and will be authored by the trial statistician and agreed by the Trial Management Group (TMG) before any unblinded data is seen.

Binary endpoints such as evidence of any new decay will be analysed by Mantel-Haenszel Chi-squared tests and odds-ratios, with the attendant 95 % confidence intervals. This type of analysis will also be carried out on safety event data. Changes in d_3_mft will be analysed by Wilcoxon tests, unless these changes are normally distributed and therefore suitable for analysis by Analysis of Covariance. Compliance will be compared with a chi-squared test. The subgroup analyses will be carried out for the primary endpoint using logistic regression with interaction terms between treatment and subgroup. All statistical tests will be two-tailed tests at the 5 % significance level. There should be no concerns regarding multiple testing. All randomised controlled trials examine secondary endpoints to examine consistency of results and to generate hypotheses for further research.

All analyses will follow the intention to treat principle [8, 11]. Study subjects will be analysed in their randomised groups regardless of the treatment actually received. Also, subjects who violate the rules of the study (‘protocol violators’) will be included in all of the analyses, provided that the 2 year endpoint data has been recorded. Ideally an intention to treat analysis would be carried out on all of the randomised subjects, but there is usually some missing primary endpoint data that prevents this. We will also carry out a ‘Worst Case Analysis’ of the Primary Endpoint. In this analysis the missing endpoints will be assumed to be treatment failures, e.g., ‘new decay’.

#### Primary efficacy analysis

The primary outcome measure is d_3_mft at two years of follow up compared to the d3mft at baseline (d_3_mft is dental decay as measured by the number of: teeth affected by decay into the dentine; missing teeth; and filled teeth). The primary analysis is the comparison between the treatment groups in percentages of children experiencing new decay as defined by the primary endpoint, namely an increase in d_3_mft.

#### Secondary efficacy analysis

Secondary analyses will be of:

- absolute change in d_3_mft at 2 years of follow up minus the d_3_mft at baseline,
- absolute change in d_3_mfs at 2 years of follow up minus the d_3_mfs at baseline.

We will do pre-defined subgroup analyses on the following types of subgroup:

- children with pre-existing disease, children without pre-existing disease,
- we will attempt to split the children into areas that are ‘extremely’ deprived and only ‘very deprived’ based on the SIMD of the child’s postcode.

#### Safety analysis

The safety data (adverse reactions) – both numbers of subjects and reactions – will be summarised by randomised group and overall using descriptive statistics. No formal statistical tests comparing the randomised groups will be pre-specified.

#### Software for statistical analysis

SAS 9.4 software for Windows, Cary, NC, USA.

#### Sample size

Assuming that prevalence data on childhood caries development in the recent literature are roughly applicable, if none of the 3 year olds with existing disease had ‘worsened’, then 24.65 % (57.63–32.98 %) would have experienced new decay. If all of the 5 year olds with decay had worsened then 57.63 % could have experienced new decay. The half-way point between these minimum and maximum percentages is therefore 41.14 %. We therefore roughly estimate that 41 % of 3 year olds will experience new decay over the course of 2 years of follow-up.

In the study of older children by Skold et al. [21] it was found that 6 month treatment by fluoride varnish for 3 years reduced the development of new caries or lesions by 54.7 % in the high risk group (75 % new decay for controls versus 34 % in the varnish group). If we multiply 54.7 % by two thirds to roughly approximate the effect of following up for 2 years rather than 3 years we would get a reduction of around 36.5 % in the development of new lesions or caries.

A 36.5 % reduction from 41 % would equal 41–41%x0.365 = 26.035 %. However, the Skold study was carried out in much older children with greater levels of decay, and our treatment will be nested within a public health intervention that includes provision of fluoride toothpaste. If we took a more cautious approach to the effect size of the treatment we could compare a 41 % worsening in the control group to a 31 % worsening in the treatment group (a 24 % reduction).

A two group chi-squared test with a two-sided significance level of 0.05 will have 90 % power to detect the difference between a Group 1 proportion of 0.41 and a Group 2 proportion of 0.31 (an odds ratio of 1.55) when the sample size in each group is 483. We therefore need a total of 966 evaluable subjects. Allowing for a pessimistic dropout rate of 40 %, we would need to randomise 805 subjects in each of the two groups, giving a total number of 1610 subjects to be randomised.

#### Economic evaluation

The economic evaluation of the Protecting Teeth at 3 Study will commence in August 2014 and will initially include the three NHS Boards that will have a new intake of participants into the trial in the autumn-winter 2014/15: NHS Fife, NHS Lothian and NHS Tayside.

##### Economic evaluation perspective

This economic evaluation will be conducted from the UK NHS perspective. The incremental costs and benefits of the fluoride varnish intervention over and above TAU only will be reported.

##### Economic evaluation data sources

Trial-related NHS resource use will be estimated using the information from direct enquiries to the trial coordinators, and from a staff costs questionnaire (Additional file 9). The trial coordinators in each participating health board will be asked to provide a list of consumables, reusable and disposable items costs (which are identical per individual child in the intervention/control group). The coordinators will also be asked to provide information on the job band of each of their PTat3 Study NHS staff. For each trial-related visit to a participating nursery the trial staff will fill in a staff costs questionnaire, which contains questions on the time spent in a nursery delivering interventions, distance travelled (calculated from postcodes of the origin and destination of journeys) and the number and type of vehicles involved in a visit to the nursery.

The previously described combined outcome/QoL questionnaire tool (see Chapter 4.2.2) will be used in order to assess the oral/general health related quality of life of the child-participants. The CHU9D will allow the calculation of QALYs, while the other outcome questionnaires will provide data on oral/general health related quality of life scores only.

##### Economic evaluation methods

The incremental costs and benefits of the fluoride varnish intervention over and above TAU only will be explored. The relationship between the general health and oral health related quality of life measures and the d_3_mft/d_3_mfs outcomes will be assessed using linear regression methods. A CAU will be also performed: with the outcome measure being QALYs, calculated using responses to the CHU9D questionnaires.

Both costs and health outcomes will be discounted at the same annual rate of 1.5 % as per the public health reference case [14].

Sensitivity analysis will be performed by varying such parameters as the costs of labour (via changing the skill mix of the intervention team by combining staff members of various job bands) and the time the trial staff spent to deliver the interventions to each child. The results of the economic evaluation will be reported via a cost-effectiveness plane and cost-effectiveness acceptability curve (CEAC) using currently accepted values of willingness to pay thresholds for a QALY (according to the National Institute for Health and Care Excellence (NICE) guidelines, [14, 28]).

#### Management and delivery

The RCB, part of the Glasgow Clinical Trials Unit, a fully registered UK CRN Clinical Trials Unit, will manage the trial data. All statistical analyses will be conducted according to the SAP specified above.

### Trial closure/Definition of end of trial

The trial will end when the TMG agrees that one or more of the following situations applies;

- last participant last study visit,
- there is insufficient funding to support further recruitment, and no reasonable prospect of additional support being obtained,
- new information makes it inappropriate to continue to randomise participants to one or other arm of the trial,
- recruitment is so poor that completion of the trial cannot reasonably be anticipated.

Within 90 days of end of trial, the Chief Investigator will submit the Declaration of End of a Clinical Trial documentation to the MHRA and Ethics Committee. The declaration of the end of a clinical trial form is available from EudraCT: European Clinical Trials website (https://eudract.ema.europa.eu/docs/forms/Declaration_Of_The_End_Of_Trial.doc). The submission of the end of trial study final report to the MHRA and Ethics Committee should be made by the Chief Investigator within 12 months of trial closure. The Sponsor should be notified that the Declaration of End of a Clinical Trial and End of Study final report has been made to the relevant bodies.

### Data handling

There are three distinct phases to study data management.

- Phase 1. Identification and consenting of study participants.
- Phase 2. Dental examinations and randomisation
- Phase 3. Intervention (varnish/control) activity recording.

#### Randomisation

The central randomisation facility at the Robertson Centre for Biostatistics, University of Glasgow (interactive voice response system, IVRS) will allocate children to each study arm. A central unblinding facility at the Robertson Centre will also be available by telephone. Notification of any unblinding will be sent to the Chief Investigator.

#### Case report forms/Electronic data record

Paper case report forms (CRFs) will be used to collect study data. Access to CRFs will be restricted, and they will be delivered by hand to the Robertson Centre by study personnel, with only authorised site-specific personnel able to make entries or amendments to their participants’ data. It is the investigator's responsibility to ensure completion and to review and approve all data captured in the CRFs.

All data handling procedures will be detailed in a Study Specific Data Management Plan. Data will be validated at the point of entry into the CRF and at regular intervals during the study. Data discrepancies will be flagged to the study site and any data changes will be recorded in order to maintain a complete audit trail (reason for change, date change made, who made change).

#### Record retention

To enable evaluations and/or audits from regulatory authorities, the investigator agrees to keep records, including the identity of all participating subjects (sufficient information to link records), all original signed informed consent forms, serious adverse event forms, source documents, and detailed records of treatment disposition in accordance with ICH GCP, local regulations, or as specified in the Clinical Study Agreement, whichever is longer. Data will be retained at the Data Centre for a minimum of 5 years.

### Trial management

#### Routine management of trial: Trial Management Group

The trial will be coordinated from Community Oral Health, Glasgow Dental School by the TMG. The TMG will include those individuals responsible for the day-to-day management of the trial, such as the Chief Investigator, statistician, trial manager, research nurse, data manager. The role of the group is to monitor all aspects of the conduct and progress of the trial, ensure that the protocol is adhered to and take appropriate action to safeguard participants and the quality of the trial itself.

Invitations have been made to a number of individuals to sit on the Study Steering Committee, to meet twice a year. Working groups within each participating Health Boards have been set up, consisting of NHS and University representatives.

#### Study monitoring

Study Monitoring Visits will be conducted by NHS Greater Glasgow and Clyde Monitor(s). The level of monitoring will be based on the outcome of the completed monitoring risk assessment; however, the minimum requirement per site will be an initiation visit following the issue of all approvals, and prior to the start of recruitment; a full monitoring visit when participants have been randomised; and a close out visit at each site after the last participant has completed the last visit. All Informed Consent Forms will be reviewed; a minimum of 10 % of subjects will be reviewed for Source Data Verification; these will be chosen at random and will consist of both subjects with reported SARs and those without any reported SARs. Additionally, the monitors will aim to review all SARs reported throughout the study.

Prior to commencement of the trial a Monitoring Plan will be written by the monitors and approved by the Sponsor’s Governance Manager.

### Protocol amendments

Any change in the study protocol will require an amendment. Any proposed protocol amendments will be initiated by the Chief Investigator and any required amendment forms will be submitted to the regulatory authority, ethics committee and sponsor. The Chief Investigator will liaise with study sponsor to determine whether an amendment is non-substantial or substantial. All amended versions of the protocol will be signed by the Chief Investigator and Sponsor representative. Before the amended protocol can be implemented favourable opinion/approval must be sought from the original reviewing REC, MHRA and Research and Development (R&D) office(s).

### Ethical considerations

#### Ethical conduct of the study

The study will be carried out in accordance with the World Medical Association Declaration of Helsinki (1964) and its revisions (Tokyo 1975, Venice 1983, Hong Kong 1989, South Africa 1996, Edinburgh 2000, and Seoul 2008). Favourable ethical opinion will be sought from The West of Scotland Research Ethics Committee 1 before participants are entered into this clinical trial. Study participants will only be allowed to enter the study once either a parent or legal guardian has provided written informed consent. The Chief Investigator will be responsible for updating the Ethics committee of any new information related to the study.

#### Informed consent

Written informed consent will be obtained from a parent or legal guardian, as applicable, of each trial participant. A participant information sheet and consent form/contraindications check will be distributed via the nursery school 2–3 weeks in advance of the consent visit and/or at least 24 h prior to consent being sought on a face-to-face basis, when a study team member present at the nursery school will explain the exact nature of the study, answer any questions and address any concerns. This explanation will include the known side-effects that may be experienced, and the risks of participating in this clinical trial. Trial participants will be informed that they are free to withdraw their consent from the study or study treatment at any time. Parents can return consent/contraindications check information to the nursery schools or at the face-to-face consent visit.

### Insurance and indemnity

The Protecting Teeth At 3 Study is co-sponsored by NHS Greater Glasgow & Clyde and The University of Glasgow. The Co-sponsors will be liable for negligent harm caused by the design of the trial. NHS indemnity is provided under the Clinical Negligence and Other Risks Indemnity Scheme (CNORIS). As the substantive employer of the Chief Investigator and as co-sponsor of the The Protecting Teeth At 3 Study, The University of Glasgow also has insurance with Royal and Sun Alliance. It will be confirmed prior to the trial starting that insurance cover will be provided automatically under the current policy. The insurance cover will be subject to NHS indemnity being in place and Ethics Committee approval being obtained.

The NHS has a duty of care to participants treated, whether or not the participant is taking part in a clinical trial, and the NHS remains liable for clinical negligence and other negligent harm to participants under its duty of care.

As this is a clinician-led study there are no arrangements for no-fault compensation.

### Funding

This study is being funded as part of the evaluation programme built in to the Childsmile programme, and is therefore funded by the Scottish Government independently of any grant awarding process.

### Co-sponsor responsibilities

Prior to study initiation, a non-commercially funded clinical trial co-sponsorship agreement will be put in place between NHS Greater Glasgow & Clyde and The University of Glasgow. The roles and liabilities each organisation will take under The Medicines for Human Use (Clinical Trials) Regulations, 2004 SI 2001:1031 are laid out in this agreement signed by both organisations. The University of Glasgow shall be responsible for carrying out the obligations and responsibilities set out in the aforementioned agreement, and shall be deemed “sponsor” for the purposes of Part 3 of the regulations in relation to the study. NHS Greater Glasgow & Clyde shall be responsible for carrying out the responsibilities set out in the agreement, and shall be deemed “sponsor” for the purposes of Parts 4, 5, 6 and 7 of the Regulations in relation to the study.

### Annual reports

Annual reports will be submitted to the ethics committee, regulatory authority and sponsor with the first submitted 1 year after the date that all trial related approvals are in place.

### Dissemination of findings

Study findings for the study will be reported through the six-monthly internal reports generated by the Childsmile Central Evaluation and Research Team. Study team members will collaborate on the production of papers to be submitted to peer-reviewed journals and abstracts of proposed presentations at national and international conferences and symposia.

## Additional files

Additional file 1:
**MHRA Notification of trial.** (PDF 53 kb) Additional file 2:
**CHU9D questionnaire – Nursery version. **(DOCX 79 kb) Additional file 3:
**PedsQL questionnaire – Nursery version. **(DOCX 129 kb) Additional file 4:
**SOHO-5 questionnaire.** (DOCX 48 kb) Additional file 5:
**Child’s use of health and dental care services questionnaire.** (DOCX 20 kb) Additional file 6:
**Subjective global transition judgement questions.** (DOCX 24 kb) Additional file 7:
**Questionnaire Cover Sheet – Round 1.** (DOCX 27 kb) Additional file 8:
**Flowchart for Assessing and Reporting Adverse Reactions.** (DOCX 38 kb) Additional file 9:
**Trial staff costs questionnaire.** (DOCX 445 kb)

## Abbreviations

CHU9D: Child Health Utility 9D
CI: contraindication
d_3_mfs: decayed missing and filled surfaces
d_3_mft: decayed missing and filled teeth
EDDN: extended duty dental nurse
IVRS: interactive voice response system
NDIP: National Dental Inspection Programme
NHS: National Health Service
PedsQL: Paediatric Quality of Life Inventory
PIN: Personal Identification Number
PIS: participant information sheet
PT@3 Study: Protecting Teeth@3 Study
QALY: Quality Adjusted Life Year
RCT: randomised controlled trial
SIMD: Scottish Index of Multiple Deprivation
SOHO-5: Scale of Oral Health Outcomes for 5-year-old children
TAU: treatment as usual
