# Supplementary material for: Comparison of the caries-protective effect of fluoride varnish with treatment as usual in nursery school attendees receiving preventive oral health support through the Childsmile oral health improvement programme — the Protecting Teeth@3 Study: a randomised controlled trial
Source: BMC Oral Health. 2015 Dec 18;15:160. doi: 10.1186/s12903-015-0146-z (PMC4683783; doi:10.1186/s12903-015-0146-z)
Supplement: Additional file 2: — CHU9D questionnaire – Nursery version. (DOCX 79 kb) [file 12903_2015_146_MOESM2_ESM.docx]

**Additional file 2: CHU9D questionnaire – Nursery version**


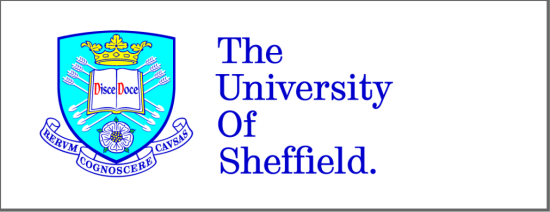


Child Health Utility 9D

Instructions

These questions ask about how your child is **today.** For each question, read all the choices and decide which one is most like your child **today**.

Then put a tick in the box next to it like this 🗹. Only tick **one** box for each question. Some questions have extra guidance with them as your child is under 5 years of age.

Example

Today my child feels quite upset so I will tick this box.

**Upset**

🞏 My child doesn’t feel upset today

🞏 My child feels a little bit upset today

🞏 My child feels a bit upset today

🗹 My child feels quite upset today

🞏 My child feels very upset today

**Now think about and answer the rest of the questions below**

1. **Worried**

🞏 My child doesn’t feel worried today

🞏 My child feels a little bit worried today

🞏 My child feels a bit worried today

🞏 My child feels quite worried today

🞏 My child feels very worried today

1. **Sad**

🞏 My child doesn’t feel sad today

🞏 My child feels a little bit sad today

🞏 My child feels a bit sad today

🞏 My child feels quite sad today

🞏 My child feels very sad today

1. **Pain**

🞏 My child doesn’t have any pain today

🞏 My child has a little bit of pain today

🞏 My child has a bit of pain today

🞏 My child has quite a lot of pain today

🞏 My child has a lot of pain today

1. **Tired**

🞏 My child doesn’t feel tired today

🞏 My child feels a little bit tired today

🞏 My child feels a bit tired today

🞏 My child feels quite tired today

🞏 My child feels very tired today

1. **Annoyed**

🞏 My child doesn’t feel annoyed today

🞏 My child feels a little bit annoyed today

🞏 My child feels a bit annoyed today

🞏 My child feels quite annoyed today

🞏 My child feels very annoyed today

1. **Nursery activities (colouring, looking at books/reading, and concentrating, as appropriate for their age)**

*If your child is at preschool/nursery/kindergarten then please think about that. If your child didn’t go today because of their health and they usually would have, please tick the last option “My child can’t do their nursery activities today”. If today is not a day they usually would have gone, then please think about how you think they would have been had they gone. If your child does not go to preschool/nursery/kindergarten, then please think about whether they have had any problems with activities such as colouring, looking at books/reading, and concentrating, as appropriate for their age.*

🞏 My child has no problems with their nursery activities today

🞏 My child has a few problems with their nursery activities today

🞏 My child has some problems with their nursery activities today

🞏 My child has many problems with their nursery activities today

🞏 My child can’t do their nursery activities today

1. **Sleep**

🞏 Last night my child had no problems sleeping

🞏 Last night my child had a few problems sleeping

🞏 Last night my child had some problems sleeping

🞏 Last night my child had many problems sleeping

🞏 Last night my child couldn’t sleep at all

1. **Daily routine (things like eating, having a bath/shower, getting dressed)**

*Please think about this question in terms of eating, drinking, toileting, washing and teeth cleaning, as appropriate for their age.*

🞏 My child has no problems with their daily routine today

🞏 My child has a few problems with their daily routine today

🞏 My child has some problems with their daily routine today

🞏 My child has many problems with their daily routine today

🞏 My child can’t do their daily routine today

1. **Able to join in activities (things like playing out with their friends, doing sports, joining in things)**

*Please think about this question in terms of the activities your child would usually be doing today.*

🞏 My child can join in with any activities today

🞏 My child can join in with most activities today

🞏 My child can join in with some activities today

🞏 My child can join in with a few activities today

🞏 My child can join in with no activities today

1. How would you rate your child’s health today?

🞏 excellent

🞏 very good

🞏 good

🞏 fair

🞏 poor

1. Do you feel there is any aspect of your child's health related quality of life that is not covered by these questions?

_______________________________________________________________________________________________________________________________________________________________________________________________________________________________________________________________________________________________________

**CHU9D questionnaire – Primary school version**


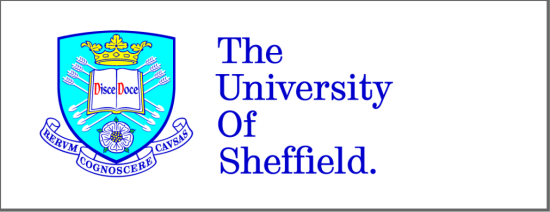


Child Health Utility 9D

Instructions

These questions ask about how your child is **today.** For each question, read all the choices and decide which one is most like your child **today**.

Then put a tick in the box next to it like this 🗹. Only tick **one** box for each question. Some questions have extra guidance with them as your child is under 5 years of age.

Example

Today my child feels quite upset so I will tick this box.

**Upset**

🞏 My child doesn’t feel upset today

🞏 My child feels a little bit upset today

🞏 My child feels a bit upset today

🗹 My child feels quite upset today

🞏 My child feels very upset today

**Now think about and answer the rest of the questions below**

1. **Worried**

🞏 My child doesn’t feel worried today

🞏 My child feels a little bit worried today

🞏 My child feels a bit worried today

🞏 My child feels quite worried today

🞏 My child feels very worried today

1. **Sad**

🞏 My child doesn’t feel sad today

🞏 My child feels a little bit sad today

🞏 My child feels a bit sad today

🞏 My child feels quite sad today

🞏 My child feels very sad today

1. **Pain**

🞏 My child doesn’t have any pain today

🞏 My child has a little bit of pain today

🞏 My child has a bit of pain today

🞏 My child has quite a lot of pain today

🞏 My child has a lot of pain today

1. **Tired**

🞏 My child doesn’t feel tired today

🞏 My child feels a little bit tired today

🞏 My child feels a bit tired today

🞏 My child feels quite tired today

🞏 My child feels very tired today

1. **Annoyed**

🞏 My child doesn’t feel annoyed today

🞏 My child feels a little bit annoyed today

🞏 My child feels a bit annoyed today

🞏 My child feels quite annoyed today

🞏 My child feels very annoyed today

1. **School Work / Homework (such as reading, writing, doing lessons)**

🞏 My child has no problems with their schoolwork / homework today

🞏 My child has a few problems with their schoolwork / homework today

🞏 My child has some problems with their schoolwork / homework today

🞏 My child has many problems with their schoolwork / homework today

🞏 My child can’t do their schoolwork / homework today

1. **Sleep**

🞏 Last night my child had no problems sleeping

🞏 Last night my child had a few problems sleeping

🞏 Last night my child had some problems sleeping

🞏 Last night my child had many problems sleeping

🞏 Last night my child couldn’t sleep at all

1. **Daily routine (things like eating, having a bath / shower, getting dressed)**

*Please think about this question in terms of eating, drinking, toileting, washing and teeth cleaning, as appropriate for their age.*

🞏 My child has no problems with their daily routine today

🞏 My child has a few problems with their daily routine today

🞏 My child has some problems with their daily routine today

🞏 My child has many problems with their daily routine today

🞏 My child can’t do their daily routine today

1. **Able to join in activities (things like playing out with their friends, doing sports, joining in things)**

*Please think about this question in terms of the activities your child would usually be doing today.*

🞏 My child can join in with any activities today

🞏 My child can join in with most activities today

🞏 My child can join in with some activities today

🞏 My child can join in with a few activities today

🞏 My child can join in with no activities today

1. How would you rate your child’s health today?

🞏 excellent

🞏 very good

🞏 good

🞏 fair

🞏 poor

1. Do you feel there is any aspect of your child's health related quality of life that is not covered by these questions?

__________________________________________________________________________________________________________________________________________________________________________________________________________________________________________________________________________________________________________________________________________________________________
