# Supplementary material for: Comparison of the caries-protective effect of fluoride varnish with treatment as usual in nursery school attendees receiving preventive oral health support through the Childsmile oral health improvement programme — the Protecting Teeth@3 Study: a randomised controlled trial
Source: BMC Oral Health. 2015 Dec 18;15:160. doi: 10.1186/s12903-015-0146-z (PMC4683783; doi:10.1186/s12903-015-0146-z)
Supplement: Additional file 3: — PedsQL questionnaire – Nursery version. (DOCX 129 kb) [file 12903_2015_146_MOESM3_ESM.docx]

**Additional file 3: PedsQL questionnaire – Nursery version**

**PedsQL**

™

Pediatric Quality of Life

Inventory

Version 4.0

**PARENT REPORT** for **TODDLERS** (ages **2-4**)

**DIRECTIONS**

On the following page is a list of things that might be a problem for **your child**. Please tell us **how much of a problem** each one has been for **your child** during the **past ONE month** by circling:

**0** if it is **never** a problem

**1** if it is **almost never** a problem

**2** if it is **sometimes** a problem

**3** if it is **often** a problem

**4** if it is **almost always** a problem

There are no right or wrong answers.

If you do not understand a question, please ask for help.

Copyright © 1998 JW Varni, Ph.D. All rights reserved

Not to be reproduced without permission

PedsQL 4.0 - Parent (2-4)

01/00

PedsQL-4.0-Core – US/English

*In the past* ***ONE month,*** *how much of a* ***problem*** *has your child had with …*

| **PHYSICAL FUNCTIONING *(problems with…)*** | **Never** | **Almost**  **Never** | **Some-**  **times** | **Often** | **Almost**  **Always** |
| --- | --- | --- | --- | --- | --- |
| 1. Walking | 0 | 1 | 2 | 3 | 4 |
| 2. Running | 0 | 1 | 2 | 3 | 4 |
| 3. Participating in active play or exercise | 0 | 1 | 2 | 3 | 4 |
| 4. Lifting something heavy | 0 | 1 | 2 | 3 | 4 |
| 5. Bathing | 0 | 1 | 2 | 3 | 4 |
| 6. Helping to pick up his or her toys | 0 | 1 | 2 | 3 | 4 |
| 7. Having hurts or aches | 0 | 1 | 2 | 3 | 4 |
| 8. Low energy level | 0 | 1 | 2 | 3 | 4 |

| **EMOTIONAL FUNCTIONING *(problems with…)*** | **Never** | **Almost**  **Never** | **Some-**  **times** | **Often** | **Almost**  **Always** |
| --- | --- | --- | --- | --- | --- |
| 1. Feeling afraid or scared | 0 | 1 | 2 | 3 | 4 |
| 2. Feeling sad or blue | 0 | 1 | 2 | 3 | 4 |
| 3. Feeling angry | 0 | 1 | 2 | 3 | 4 |
| 4. Trouble sleeping | 0 | 1 | 2 | 3 | 4 |
| 5. Worrying | 0 | 1 | 2 | 3 | 4 |

| **SOCIAL FUNCTIONING *(problems with…)*** | **Never** | **Almost**  **Never** | **Some- times** | **Often** | **Almost**  **Always** |
| --- | --- | --- | --- | --- | --- |
| 1. Playing with other children | 0 | 1 | 2 | 3 | 4 |
| 2. Other kids not wanting to play with him or her | 0 | 1 | 2 | 3 | 4 |
| 3. Getting teased by other children | 0 | 1 | 2 | 3 | 4 |
| 4. Not able to do things that other children his or her age can do | 0 | 1 | 2 | 3 | 4 |
| 5. Keeping up when playing with other children | 0 | 1 | 2 | 3 | 4 |

****Please complete this section if your child attends nursery or daycare***

| **NURSERY / DAY CARE FUNCTIONING *(problems with…)*** | **Never** | **Almost**  **Never** | **Some- times** | **Often** | **Almost**  **Always** |
| --- | --- | --- | --- | --- | --- |
| 1. Doing the same nursery/day care activities as peers | 0 | 1 | 2 | 3 | 4 |
| 2. Missing nursery/day care because of not feeling well | 0 | 1 | 2 | 3 | 4 |
| 3. Missing nursery/day care to go to the doctor or hospital | 0 | 1 | 2 | 3 | 4 |

PedsQL 4.0 - Parent (2-4)

01/00

PedsQL-4.0-Core – US/English

Copyright © 1998 JW Varni, Ph.D. All rights reserved

Not to be reproduced without permission

**PedsQL questionnaire – Primary school version**

**PedsQL**

™

Pediatric Quality of LifeInventory

Version 4.0

**PARENT REPORT** for **TODDLERS** (ages **2-4**)

**DIRECTIONS**

On the following page is a list of things that might be a problem for **your child**. Please tell us **how much of a problem** each one has been for **your child** during the **past ONE month** by circling:

**0** if it is **never** a problem

**1** if it is **almost never** a problem

**2** if it is **sometimes** a problem

**3** if it is **often** a problem

**4** if it is **almost always** a problem

There are no right or wrong answers. If you do not understand a

question, please ask for help.

Copyright © 1998 JW Varni, Ph.D. All rights reserved

Not to be reproduced without permission

Copyright © 1998 JW Varni, Ph.D. All rights reserved

Not to be reproduced without permission

PedsQL 4.0 - Parent (2-4)

01 / 00

PedsQL-4.0-Core – US / English

PedsQL-4.0-Core-PT_eng-USori.doc

*In the past* ***ONE month,*** *how much of a* ***problem*** *has your child had with …*

| **PHYSICAL FUNCTIONING *(problems with…)*** | **Never** | **Almost**  **Never** | **Some-**  **times** | **Often** | **Almost**  **Always** |
| --- | --- | --- | --- | --- | --- |
| 1. Walking | 0 | 1 | 2 | 3 | 4 |
| 2. Running | 0 | 1 | 2 | 3 | 4 |
| 3. Participating in active play or exercise | 0 | 1 | 2 | 3 | 4 |
| 4. Lifting something heavy | 0 | 1 | 2 | 3 | 4 |
| 5. Bathing | 0 | 1 | 2 | 3 | 4 |
| 6. Helping to pick up his or her toys | 0 | 1 | 2 | 3 | 4 |
| 7. Having hurts or aches | 0 | 1 | 2 | 3 | 4 |
| 8. Low energy level | 0 | 1 | 2 | 3 | 4 |

| **EMOTIONAL FUNCTIONING *(problems with…)*** | **Never** | **Almost**  **Never** | **Some-**  **times** | **Often** | **Almost**  **Always** |
| --- | --- | --- | --- | --- | --- |
| 1. Feeling afraid or scared | 0 | 1 | 2 | 3 | 4 |
| 2. Feeling sad or blue | 0 | 1 | 2 | 3 | 4 |
| 3. Feeling angry | 0 | 1 | 2 | 3 | 4 |
| 4. Trouble sleeping | 0 | 1 | 2 | 3 | 4 |
| 5. Worrying | 0 | 1 | 2 | 3 | 4 |

| **SOCIAL FUNCTIONING *(problems with…)*** | **Never** | **Almost**  **Never** | **Some- times** | **Often** | **Almost**  **Always** |
| --- | --- | --- | --- | --- | --- |
| 1. Playing with other children | 0 | 1 | 2 | 3 | 4 |
| 2. Other kids not wanting to play with him or her | 0 | 1 | 2 | 3 | 4 |
| 3. Getting teased by other children | 0 | 1 | 2 | 3 | 4 |
| 4. Not able to do things that other children his or her age can do | 0 | 1 | 2 | 3 | 4 |
| 5. Keeping up when playing with other children | 0 | 1 | 2 | 3 | 4 |

****Please complete this section if your child attends school or daycare***

| **SCHOOL FUNCTIONING *(problems with…)*** | **Never** | **Almost**  **Never** | **Some- times** | **Often** | **Almost**  **Always** |
| --- | --- | --- | --- | --- | --- |
| 1. Doing the same school activities as peers | 0 | 1 | 2 | 3 | 4 |
| 2. Missing school / daycare because of not feeling well | 0 | 1 | 2 | 3 | 4 |
| 3. Missing school / daycare to go to the doctor or hospital | 0 | 1 | 2 | 3 | 4 |

Copyright © 1998 JW Varni, Ph.D. All rights reserved

Not to be reproduced without permission

PedsQL 4.0 - Parent (2-4)

01 / 00

PedsQL-4.0-Core – US / English

PedsQL-4.0-Core-PT_eng-USori.doc

**PedsQL questionnaire – Oral Health Module**

*In the past* ***ONE month,*** *how much of a* ***problem*** *has your child had with …*

| **ABOUT MY CHILD’S TEETH AND MOUTH**  ***(problems with…)*** | **Never** | **Almost**  **Never** | **Some-**  **times** | **Often** | **Almost**  **Always** |
| --- | --- | --- | --- | --- | --- |
| 1. Having tooth pain | 0 | 1 | 2 | 3 | 4 |
| 2. Having tooth pain when eating or drinking something hot, cold, or sweet | 0 | 1 | 2 | 3 | 4 |
| 3. Having teeth that are dark in color | 0 | 1 | 2 | 3 | 4 |
| 4. Having gum pain | 0 | 1 | 2 | 3 | 4 |
| 5. Having blood on his or her toothbrush after brushing | 0 | 1 | 2 | 3 | 4 |

PedsQL 3.0 Parent (2-4) Oral Health Not to be reproduced without permission Copyright © 1998 JW Varni, Ph.D.

06 / 08
