# Supplementary material for: Comparison of the caries-protective effect of fluoride varnish with treatment as usual in nursery school attendees receiving preventive oral health support through the Childsmile oral health improvement programme — the Protecting Teeth@3 Study: a randomised controlled trial
Source: BMC Oral Health. 2015 Dec 18;15:160. doi: 10.1186/s12903-015-0146-z (PMC4683783; doi:10.1186/s12903-015-0146-z)
Supplement: Additional file 4: — SOHO-5 questionnaire. (DOCX 48 kb) [file 12903_2015_146_MOESM4_ESM.docx]

# **Additional file 4: SOHO-5 questionnaire**

The Scale of Oral Health Outcomes for children (SOHO-5)

**Questions about your child’s dental health** and the effect of your child’s teeth on his / her daily life

Now we would like to know more about your child’s teeth and mouth and how they affect their daily life. Please **tick** the most appropriate response on the scale below.

**In the PAST 12 MONTHS has you child:**

**1.**  Had any difficulty **eating** because of his / her teeth?

| not at all | a little | moderate | a lot | a great deal | don’t know |
| --- | --- | --- | --- | --- | --- |
|  |  |  |  |  |  |

**2.** Had any difficulty **speaking** because of his / her teeth?

| not at all | a little | moderate | a lot | a great deal | don’t know |
| --- | --- | --- | --- | --- | --- |

**3.** Had any difficulty **playing** because of his / her teeth?

| not at all | a little | moderate | a lot | a great deal | don’t know |
| --- | --- | --- | --- | --- | --- |

**4.** Avoided **smiling** because of the **appearance** of his / her teeth?

| not at all | a little | moderate | a lot | a great deal | don’t know |
| --- | --- | --- | --- | --- | --- |
|  |  |  |  |  |  |

**5.** Avoided **smiling** because of the **state (holes in teeth, pain)** of his / her teeth?

| not at all | a little | moderate | a lot | a great deal | don’t know |
| --- | --- | --- | --- | --- | --- |
|  |  |  |  |  |  |

**6.** Had difficulty **sleeping** because of his / her teeth?

| not at all | a little | moderate | a lot | a great deal | don’t know |
| --- | --- | --- | --- | --- | --- |

**7.** Has your child’s **self-confidence** been affected because of his / her teeth (in the past 12 months)?

| not at all | a little | moderate | a lot | a great deal | don’t know |
| --- | --- | --- | --- | --- | --- |

# **Subjective global transition judgement questions**

**a) How did your child’s general health change in the PAST 12 MONTHS?** *(Tick one)*

| Worsened a lot | Worsened a little | Stayed the same | Improved a little | Improved a lot |
| --- | --- | --- | --- | --- |

**b) How did your child’s oral health change in the PAST 12 MONTHS?** *(Tick one)*

| Worsened a lot | Worsened a little | Stayed the same | Improved a little | Improved a lot |
| --- | --- | --- | --- | --- |
