# Supplementary material for: Comparison of the caries-protective effect of fluoride varnish with treatment as usual in nursery school attendees receiving preventive oral health support through the Childsmile oral health improvement programme — the Protecting Teeth@3 Study: a randomised controlled trial
Source: BMC Oral Health. 2015 Dec 18;15:160. doi: 10.1186/s12903-015-0146-z (PMC4683783; doi:10.1186/s12903-015-0146-z)
Supplement: Additional file 5: — Child’s use of health and dental care services questionnaire. (DOCX 20 kb) [file 12903_2015_146_MOESM5_ESM.docx]

# **Additional file 5: Child’s use of health and dental care services questionnaire**

| **YOUR CHILD’S USE OF HEALTH AND DENTAL CARE SERVICES**  **in the past 12 months** |
| --- |

**1) What health and dental care services has your child used within the PAST 12 MONTHS?**

***Note: Please enter ‘0’ (zero) if service has not been used***

| **Service** | **Total number of contacts** |
| --- | --- |
| General Practitioner (GP) |  |
| Accident and Emergency (A&E) visit |  |
| Dentist (at your family dental practice) |  |
| Dental hygienist / therapist (at your family dental practice) |  |
| Speech therapist |  |
| Hospital inpatient stay | Number of nights: _____ |
| Hospital outpatient stay |  |
| Other (1): _____________________________________ |  |
| Other (2): _____________________________________ |  |

**2) Please list below your child’s use of any medication (e.g. pain-killers, antibiotics) he / she has taken within the PAST 12 MONTHS:**

| **Name of medication** | **How long did your child take this medication for?**  (for example, ‘3 days’ or ‘1 week’) | **Daily dosage** |
| --- | --- | --- |
| 1. |  |  |
| 2. |  |  |
| 3. |  |  |
| 4. |  |  |
| 5. |  |  |

**3) In the past 12 months, approximately how many days has your child had off nursery / school due to ill health (including ill health due to dental problems)?**

Number of days off nursery / school:

**4) Did you or other parent / guardian have to take time off work or your usual daily activities due to your child being off nursery / school (due to ill health) in the past 12 months?**

*Yes No (tick one)* If yes, please state how many days:
