# Supplementary material for: Comparison of the caries-protective effect of fluoride varnish with treatment as usual in nursery school attendees receiving preventive oral health support through the Childsmile oral health improvement programme — the Protecting Teeth@3 Study: a randomised controlled trial
Source: BMC Oral Health. 2015 Dec 18;15:160. doi: 10.1186/s12903-015-0146-z (PMC4683783; doi:10.1186/s12903-015-0146-z)
Supplement: Additional file 6: — Subjective global transition judgement questions. (DOCX 24 kb) [file 12903_2015_146_MOESM6_ESM.docx]

# **Additional file 6: Subjective global transition judgement questions**

**a) How did your child’s general health change in the PAST 12 MONTHS?** *(Tick one)*

| Worsened a lot | Worsened a little | Stayed the same | Improved a little | Improved a lot |
| --- | --- | --- | --- | --- |

**b) How did your child’s oral health change in the PAST 12 MONTHS?** *(Tick one)*

| Worsened a lot | Worsened a little | Stayed the same | Improved a little | Improved a lot |
| --- | --- | --- | --- | --- |
