# Supplementary material for: Comparison of the caries-protective effect of fluoride varnish with treatment as usual in nursery school attendees receiving preventive oral health support through the Childsmile oral health improvement programme — the Protecting Teeth@3 Study: a randomised controlled trial
Source: BMC Oral Health. 2015 Dec 18;15:160. doi: 10.1186/s12903-015-0146-z (PMC4683783; doi:10.1186/s12903-015-0146-z)
Supplement: Additional file 7: — Questionnaire Cover Sheet – Round 1. (DOCX 27 kb) [file 12903_2015_146_MOESM7_ESM.docx]

**Additional file 7: Questionnaire Cover Sheet – Round 1**

**(R1)**

**How does a child’s health affect their quality of life?**

We would like to ask you a series of questions to find out how a child’s oral and general health affects their quality of life. The questionnaire takes around 10 to 15 minutes to fill in. Some questions might seem to cover the same ground, but they do serve slightly different purposes, so please answer all questions in this questionnaire pack.

Like your child’s involvement in the main Protecting Teeth @ 3 Study, participation in this survey is voluntary. If you decide not to complete the questionnaire, your decision will not affect your child’s continued participation in the study, or the standard of care you or your child may receive from the NHS, now or in the future. Your answers will be treated confidentially.

We would like you to fill in the questionnaire three times - now, in a year’s time, and at the end of the study in two years’ time. This will help us to assess if there have been any changes over time. If you would like more information you can contact us on: 0141 211 9853 or 0141 211 9802.

**PLEASE ANSWER ALL QUESTIONS IN THIS PACK, THEN RETURN IT IN THE PRE-PAID ENVELOPE PROVIDED OR HAND IT IN TO YOUR CHILD’S NURSERY FOR COLLECTION.**

***As a thank you for helping us, we are offering you the chance to enter a free prize draw to win an iPad. Each time you fill in a questionnaire you have a chance of winning, so even if you win the first time you will still be entered into the next two draws. Please tick the most appropriate box below:***

**Yes,** I would like to take part in the draw

**No**, I do not want to take part in the draw

**In order to be included in the draw you must return your completed questionnaires within 28 days.**

| **OFFICE USE ONLY** | |
| --- | --- |
| Participant’s ID: | |
| Date received: | Date entered onto database: |
| *Received by (initials):* | *Entered by (initials):* |

Version 1.0 30/07/2014

**Questionnaire Cover Sheet – Round 2**

**(R2)**

**How does a child’s health affect their quality of life?**

We would like to ask you the same series of questions as we asked you a year ago, in order to find out how a child’s oral and general health affect their quality of life. The questionnaire takes around 10 to 15 minutes to fill in. Some questions might seem to cover the same ground, but they do serve slightly different purposes, so please answer all questions in this questionnaire pack.

Like your child’s involvement in the main Protecting Teeth @ 3 Study, participation in this survey is voluntary. If you decide not to complete the questionnaire, your decision will not affect your child’s continued participation in the study, or the standard of care you or your child may receive from the NHS, now or in the future. Your answers will be treated confidentially.

This is the second round of this questionnaire, out of a series of three in total. Even though you filled a similar questionnaire about a year ago, we need you to fill in this one as well to help us to assess if there have been any changes over time. You will also receive the final questionnaire in a year’s time.

If you would like more information you can contact us on: 0141 211 9853 or 0141 211 9802.

**PLEASE ANSWER ALL QUESTIONS IN THIS PACK, THEN POST IT BACK TO US**

**IN THE PRE-PAID ENVELOPE PROVIDED.**

***As a thank you for helping us, we are offering you the chance to enter a free prize draw to win an iPad. Each time you fill in a questionnaire you have a chance of winning. Please tick the most appropriate box below:***

**Yes,** I would like to take part in the draw

**No**, I do not want to take part in the draw

**In order to be included in the draw you must return your completed questionnaires within 28 days.**

| **OFFICE USE ONLY** | |
| --- | --- |
| Participant’s ID: | |
| Date received: | Date entered onto database: |
| *Received by (initials):* | *Entered by (initials):* |

Version 1.1 03/08/2015 **Questionnaire Cover Sheet – Round 3**

**(R3)**

**How does a child’s health affect their quality of life?**

We would like to ask you the same series of questions as we asked you a year and two years ago, in order to find out how a child’s oral and general health affect their quality of life. The questionnaire takes around 10 to 15 minutes to fill in. Some questions might seem to cover the same ground, but they do serve slightly different purposes, so please answer all questions in this questionnaire pack.

Like your child’s involvement in the main Protecting Teeth @ 3 Study, participation in this survey is voluntary. If you decide not to complete the questionnaire, your decision will not affect your child’s continued participation in the study, or the standard of care you or your child may receive from the NHS, now or in the future. Your answers will be treated confidentially.

This is the third and final round of this questionnaire. Even though you may have filled earlier versions, we need you to fill in this one as well to help us to assess if there have been any changes over time.

If you would like more information you can contact us on: 0141 211 9853 or 0141 211 9802.

**PLEASE ANSWER ALL QUESTIONS IN THIS PACK, THEN POST IT BACK TO US**

**IN THE PRE-PAID ENVELOPE PROVIDED.**

***As a thank you for helping us, we are offering you the chance to enter a free prize draw to win an iPad. Each time you fill in a questionnaire you have a chance of winning. Please tick the most appropriate box below:***

**Yes,** I would like to take part in the draw

**No**, I do not want to take part in the draw

**In order to be included in the draw you must return your completed questionnaires within 28 days.**

| **OFFICE USE ONLY** | |
| --- | --- |
| Participant’s ID: | |
| Date received: | Date entered onto database: |
| *Received by (initials):* | *Entered by (initials):* |

Version 1.2 03/08/201
