# Supplementary material for: Comparison of the caries-protective effect of fluoride varnish with treatment as usual in nursery school attendees receiving preventive oral health support through the Childsmile oral health improvement programme — the Protecting Teeth@3 Study: a randomised controlled trial
Source: BMC Oral Health. 2015 Dec 18;15:160. doi: 10.1186/s12903-015-0146-z (PMC4683783; doi:10.1186/s12903-015-0146-z)
Supplement: Additional file 8: — Flowchart for Assessing and Reporting Adverse Reactions. (DOCX 38 kb) [file 12903_2015_146_MOESM8_ESM.docx]

# **Additional file 8:** **Flowchart for Assessing and Reporting Adverse Reactions**

*(applies only to events which are deemed to be, or suspected of being, related)*

|  |  | **Event detected at study visit** |  | **Event suspected by parent/guardian** |  |  |  |
| --- | --- | --- | --- | --- | --- | --- | --- |
|  |  |  |  |  |  |  |  |
|  |  |  |  | **Childsmile or Fluoride varnish aftercare leaflets (phone numbers)** |  |  |  |
|  |  |  |  |  |  |  |  |
| **Assess seriousness, severity** |  |  | **Childsmile Study Staff** |  |  |  |  |
|  |  |  |  |  |  |  |  |
|  |  |  |  |  |  |  |  |
|  |  |  |  |  |  |  |  |
|  |  |  |  |  |  | **Case Report Form** | **If serious, SAE report** |
|  |  |  |  |  |  |  |  |
|  |  |  |  |  |  |  |  |
|  |  |  |  |  |  |  |  |
| **Assess relatedness and severity. Re-assess seriousness** |  |  | **Principal Investigator** |  |  |  |  |
|  |  |  |  |  |  |  |  |
|  |  |  |  |  |  |  |  |
|  |  |  |  |  |  |  | **Pharmaco - vigilance office** |
|  |  |  |  |  |  |  |  |
|  |  |  |  |  |  |  |  |
| **Assess expectedness** |  |  | **Chief Investigator** |  |  |  | **If serious and unexpected, expedited reporting** |
|  |  |  |  |  |  |  |  |
|  |  |  |  |  |  |  |  |
|  |  |  |  |  |  |  | **MHRA, ETHICS** |
|  |  |  |  |  |  |  |  |
