# Supplementary material for: Comparison of the caries-protective effect of fluoride varnish with treatment as usual in nursery school attendees receiving preventive oral health support through the Childsmile oral health improvement programme — the Protecting Teeth@3 Study: a randomised controlled trial
Source: BMC Oral Health. 2015 Dec 18;15:160. doi: 10.1186/s12903-015-0146-z (PMC4683783; doi:10.1186/s12903-015-0146-z)
Supplement: Additional file 9: — Trial staff costs questionnaire. (DOCX 445 kb) [file 12903_2015_146_MOESM9_ESM.docx]

# **Additional file 9: Trial staff costs questionnaire**

| ­­­­­­­­­­­­­***This questionnaire should be completed by one intervention team member (ideally the team lead) on behalf of all team members present on the Fluoride Varnish / Treatment as Usual Visit.*** *(If a baseline dental inspection is happening on the same visit, do NOT include the details of the inspecting dentist and the scribe.)*  **The purpose of collecting this information is to calculate the costs incurred by the NHS in regard to the PT@3 Study and relate these to the outcomes in the participants’ d_3_mft at the end of the trial.**  **Please complete a questionnaire for each nursery school visited, even if visits occurred on the same day.**  *Please fill in the questionnaire using ink and block capitals.* |
| --- |

**Section 1**

| **Nursery name:**  ______________________________ | **Date of Visit:**  **____________________________________** |
| --- | --- |

**Section 2 – Staff travel**

**In this section we need to collect information on how *all* intervention team members travelled to and from this nursery. Where staff shared transport please list all team members who travelled together in column c) in the table below**

| **a) All staff journeys**  **to / from this nursery** | | **b) Mode of transport**  (van, car, bus, train, if other please specify) | **c) Names & Surnames of team members who shared this journey** | **Travel TO this nursery** | | **Travel FROM this nursery** | |
| --- | --- | --- | --- | --- | --- | --- | --- |
|  |  |  |  | **d) Postcode of origin of your work-related travel** | **e) Approx. mileage** | **f) Postcode of destination**  (where you are going **after** this nursery visit) | **g) Approx. mileage to this destination** |
| ***Ex1*** | **am** | *Childsmile van* | *Mary Poppins, Sue Barton* | *G2 3JZ* | *5 miles* | *G21 2DA* | *4 miles* |
|  | **pm** |  |  |  |  |  |  |
| ***Ex2*** | **am** | *Bus* | *Jane Bloggs (travelled alone)* | *G34 9HQ* | *2 miles* | *G34 9HQ* | *2 miles* |
|  | **pm** |  |  |  |  |  |  |
| **1** | **am** |  |  |  |  |  |  |
|  | **pm** |  |  |  |  |  |  |
| **2** | **am** |  |  |  |  |  |  |
|  | **pm** |  |  |  |  |  |  |
| **3** | **am** |  |  |  |  |  |  |
|  | **pm** |  |  |  |  |  |  |
| **4** | **am** |  |  |  |  |  |  |
|  | **pm** |  |  |  |  |  |  |
| **5** | **am** |  |  |  |  |  |  |
|  | **pm** |  |  |  |  |  |  |
| **6** | **am** |  |  |  |  |  |  |
|  | **pm** |  |  |  |  |  |  |
| **7** | **am** |  |  |  |  |  |  |
|  | **pm** |  |  |  |  |  |  |

**Section 3 – Intervention (Fluoride Varnish / TAU) day**

| What PT@3 activities were undertaken during **this** nursery visit? (Please **tick all** that apply):   \| □ \| Baseline dental inspections \| \| --- \| --- \| \| □ \| Randomisations \| \| □ \| Interventions (fluoride varnishing / treatment as usual) \| |
| --- | --- | --- | --- | --- | --- | --- |

| **Morning session** | **Afternoon session** |
| --- | --- |
| a) When did the first intervention team member **arrive** at the nursery (**AM**)?  ______hh ______mm  *(****Example:*** *8:40am)*  b) When all the intervention activities were completed, when did the last intervention team member **leave** the nursery (before going elsewhere)?  ______hh ______mm  *(****Example:*** *11:35am)* | c) When did the first intervention team member **arrive** at the nursery (**PM**)?  ______hh ______mm  *(****Example:*** *13:00 or 1:00pm)*  d) When all the intervention activities were completed, when did the last intervention team member **leave** the nursery?  ______hh ______mm  *(****Example:*** *14:30 or 2:30pm)* |
| **If you did not leave the nursery between the morning and afternoon sessions, indicate only one set of times: e.g. *arrived at 8:40am, left at 14:30*** | |

**Section 4 – Do you have any additional comments in relation to this nursery visit?**

|  |
| --- |

***Thank you very much!***

***Please file the completed form in the relevant nursery PT@3 folder at the end of the day***
