# Supplementary material for: Comparison of the caries-protective effect of fluoride varnish with treatment as usual in nursery school attendees receiving preventive oral health support through the Childsmile oral health improvement programme — the Protecting Teeth@3 Study: a randomised controlled trial
Source: BMC Oral Health. 2015 Dec 18;15:160. doi: 10.1186/s12903-015-0146-z (PMC4683783; doi:10.1186/s12903-015-0146-z)
Supplement: Additional file 1: — MHRA Notification of trial. (PDF 53 kb) [file 12903_2015_146_MOESM1_ESM.pdf]

Safeguarding public health

MHRA

Dr M Barber
NHS GREATER GLASGOW AND CLYDE
TENNENT BUILDING, 38 CHURCH STREET
WESTERN INFIRMARY
GLASGOW
G11 6NT
UNITED KINGDOM

12/10/2012

Dear Dr M Barber

THE MEDICINES FOR HUMAN USE (CLINICAL TRIALS) REGULATIONS 2004 S.I. 2004/1031 (as amended)(the 'Regulations')

Our Reference:	24712/0025/001-0001
Eudract Number:	2012-002287-26
Product:	DURAPHAT DENTAL SUSPENSION 50MG/ML
Protocol number:	GN12CO201

ACKNOWLEDGEMENT OF NOTIFICATION

I am writing to confirm receipt of your notification of a clinical trial received on 11/10/2012.

For the purposes of regulation 18(2)(c) or 20(2)(a) and (5) of the Regulations (as appropriate), this letter is notice of authorisation of the trial referenced above with effect from 25/10/2012 (the 'effective date') subject to the following condition:

- that no further correspondence is received from the Licensing Authority before the effective date requiring full assessment of your request for authorisation.

You are reminded that your trial may be suspended or terminated at any time by the Licensing Authority in accordance with regulation 31. You must notify the Licensing Authority within 90 days of the trial ending.

Finally, you are reminded that a favourable opinion from the Ethics Committee is also required before this trial can proceed.

Yours sincerely,

Clinical Trials Unit
MHRA
